# The relationship between cognitive schemas activated in sexual context and early maladaptive schemas among married women of childbearing age

**DOI:** 10.1186/s40359-022-00829-1

**Published:** 2022-05-23

**Authors:** Zainab Alimoradi, Simin Zarabadipour, Nicole A. Arrato, Mark D. Griffiths, Barbara L. Andersen, Nasim Bahrami

**Affiliations:** 1 Social Determinants of Health Research Center, Research Institute for Prevention of Non-Communicable Diseases, Qazvin University of Medical Sciences, Qazvin, 34197-59811 Iran; 2 Stress and Immunity Cancer Projects Lab, Department of Psychology, Ohio State University, Columbus, OH USA; 3 Psychology Department, Nottingham Trent University, Nottingham, UK

**Keywords:** Cognitive sexual schemas, Early maladaptive schemas, Iranian women, Childbearing females

## Abstract

**Background:**

Healthy sex can be affected by cognitive schemas activated in the sexual context (CSASCs) and early maladaptive schemas (EMSs). Cognitive schemas are the nuclear structure of the cognitive system which facilitate the interaction between individuals and their environments. CSASCs are emotional and behavioral responses in the sexual context. EMSs are extremely stable, enduring and are developed throughout the life of the individual, beginning in childhood. The present study investigated the relationship between CSASCs and EMSs among married women of childbearing age.

**Methods:**

In a cross-sectional study, 260 married women of childbearing age participated. Using two-stage sampling, ten comprehensive urban health centers were first randomly selected and then 26 individuals from each center were invited to participate. Data collection included demographic variables, the Young Schema Questionnaire-Short Form (YSQ-SF) with 15 EMSs (emotional deprivation, abandonment, mistrust/abuse, social alienation, defectiveness, incompetence, dependency, vulnerability to harm, enmeshment, subjugation of needs, self-sacrifice, emotional inhibition, unrelenting standards, entitlement, and insufficient self-control), and the Cognitive Schema Activation in Sexual Context Questionnaires (CSASCQ) with five subscales (undesirability/rejection, incompetence, self-depreciation, difference/loneliness, and helplessness). Data analysis was performed using a uni-variable and multi-variable linear regression model with a stepwise method at a significance level of 0.05.

**Results:**

The mean age of the participants was 32.48 years and the average duration of their marriage was 10.34 years. The average score of early maladaptive schemas on the YSQ-SF was 151.5 (out of 450). Higher scores indicate more severe maladaptive schemas, although the total score has no defined cutoff point. Scores greater than 15 on each subscale constitute the internalization of that particular maladaptive schema. The highest average subscale scores were the schemas of self-sacrifice and unrelenting standards (M = 16.12, 15.90, respectively), indicating that these two schemas play important roles in the cognition of the participants. The mean score on the CSASCQ was 34.60 (SD ± 12.59; range: 25–125), with the highest mean reported on the loneliness subscale. Hypoactive sexual desire was the most common unpleasant sexual problem (6.9%) and disillusion was the most dominant feeling experienced by participants (33.3%). The results of the multivariable model showed that the following were significant predictors of the CSASC: three subscales of EMS (emotional deprivation [β = 0.28], social isolation [β = 0.31], and emotional inhibition [β = 0.14]) and two demographic variables (job [β = 0.11] and consanguineous marriage [β = 0.20]). In total, the multi-variable model explained 92% of variance of CSASCs.

**Conclusions:**

The present study found a significant and meaningful association between EMSs and CSASCs adjusting for the effect of socio-demographic characteristics. The findings indicate that the study of schemas and schema therapy should be considered in both premarital and marital counseling.

## Background

A schema may be defined as a cognitive structure that includes stable beliefs and assumptions about the self, others, and the world, and functions as a broad organizing principle that directs an individual’s cognitive processing in their life events [1, 2]. Schemas can play an important role in the quality of various aspects of marital relationships [3]. Self-schemas are cognitive generalizations about the self. Schemas can also impact relationships, and an individual’s cognitive schemas have been shown to be relevant in studies on the causes of divorce [4]. Young and Gluhoski found that if there are inconsistent schemas between spouses, the disconnect can lead to dissatisfaction and eventually divorce [5].

Based on Young’s work, early maladaptive schemas (EMSs) are said to consist of deep and pervasive patterns or themes formed by negative memories, emotions, cognitions, and bodily sensations. Maladaptive schemas are often formed in childhood or adolescence, and as they persist throughout the course of life, they can cause inadequacy in individuals’ relationships with themselves and others [2, 6]. When maladaptive schemas are activated in the face of a challenging situation, this leads to dysfunctional perceptions, thinking patterns, and behaviors that may eventually lead to psychiatric problems, such as anxiety or depressive disorders, eating disorders, and even personality disorders [7].

Maladaptive schemas often produce negative bias in interpreting events, which may lead to problems in couples’ emotional relationships [8]. In the field of sexual function, schemas include assumptions and expectations about the spouse and the marital relationship [9]. In this regard, it has been shown that maladaptive schemas are associated with low marital satisfaction [8]. A relationship has also been found between maladaptive schemas and difficulties with individuals satisfying their own sexual needs, especially sexual needs, which can eventually lead to sexual dissatisfaction [10]. A study by Oliveira and Nobreh showed that sexual dissatisfaction and reduced marital satisfaction were significantly associated with early maladaptive schemas [11].

In addition to general schemas which constitute of stable beliefs and assumptions about the general aspects of life [1, 2], individuals also have sexual self-schemas. Sexual self-schemas are cognitive generalizations regarding sexual aspects of the self that represent a core component of an individual’s sexuality [12]. An individual’s sexual beliefs and attitudes also influence the processing of sexual information and guiding of sexual behavior [13]. Moreover, their attitudes or knowledge about sex or their ability to have sex are important factors affecting sexual activity and satisfaction. Therefore, individuals’ thoughts, attitudes, and values are important components of sexual function and are sources of influence on sexual response and satisfaction [14, 15]. Like maladaptive schemas which often produce negative bias in interpreting events [7], unpleasant sexual experiences might be associated with negative sexual schemas [16]. Individuals may develop specific, faulty cognitive constructions (i.e., cognitive vulnerabilities) that predispose them to develop specific psychopathological syndromes [17]. In this regard, self-schemas activated by an individual when facing sexual failure situations are called cognitive schemas activated in sexual context [18].

Overall, early maladaptive schemas have a detrimental effect on an individual’s understanding of various situations, including marital life and sexual relationships [19]. Moreover, healthy sexual relations in marital life (such as expressing feelings and emotions to one’s spouse and engaging in sexual intercourse) can influence the quality of the marital relationship, sexual satisfaction, and each partner’s sexual schemas [20]. Therefore, healthy sexual relationships can be affected by both cognitive schemas activated in sexual context and early maladaptive schemas [21, 22]. Moreover, it should be noted that the impact of contextual variables such as demographic and social characteristics on various sexual issues has been shown in several studies. Variables such as age [24], education [24], job [23, 25], economic status [26], and type of marriage [24, 26] have been correlated with sexual variables in various studies.

Most studies related to schemas have been carried out with specific groups of participants [27–29] or have focused on the impact of early maladaptive schemas on various aspects of married life (e.g., marital satisfaction, sexual satisfaction) [23, 30–32]. Although the relationship between early cognitive schemas and sexually activated schemas has been investigated in various studies, to the best of the authors’ knowledge, the relationship between cognitive schemas activated in a sexual context and early maladaptive schemas has not been considered in an Iranian context. Therefore, the present study sought to fill this gap.

Due to the fact that sexual activity in Iranian society is legitimate only in the context of marriage, all participants in this study were married women. Marriage in Iranian society is traditional and individuals sometimes do not play an active role in choosing their sexual partner as a spouse. In Iranian society, the culture of sexual relationship is a patriarchal culture. Most women have a passive role in the relationship; they often feel that they should not initiate a relationship and they should use different ways to keep their sexual partner (i.e., their husband) satisfied. Understanding schemas and the relationships between different schemas can be an effective step in obtaining insight to design and implement culturally-based schematic interventions within marital counseling. Therefore, the aim of the present study was to investigate the relationship between cognitive schemas activated in sexual context and early maladaptive schemas among married women of childbearing age in an Iranian sample.

## Methods

### Design and participants

A cross-sectional study was carried out in ten comprehensive health centers in Qazvin with the participation of 260 married women of childbearing age. Qazvin is the largest city and capital of the Province of Qazvin in Iran. Its population is approximately 650,000 and is located 150 km northwest of Tehran. Comprehensive health centers provide community-based health services to women based on their age and health status including preconception counseling, prenatal care, postpartum care, monitoring children’s growth and development, and breast examination. They are located in different parts of the city and are therefore the best centers to recruit women from different social, economic, and cultural classes. The exclusion criteria were: currently pregnant or lactating, premenopausal or menopausal age, having other known medical or psychological illness based on participant self-report, currently experiencing a stressful event (e.g., recent loss of a loved one), and/or unwillingness to participate in the study.

### Sample size estimation

No relevant study was found in this field, so it was not possible to model the present study’s sample size with a twin study. Considering the minimum correlation coefficient of 0.15, α = 0.05, and β = 0.20, the required sample size was estimated to be 260 individuals.

### Sampling procedure

To recruit a research sample with maximum diversity in terms of economic, social, and cultural status, two-stage cluster sampling was used. In the first stage, two health centers were randomly selected from each of the five areas of Qazvin, with each area considered to be a cluster. Then, 26 individuals from each of the selected health centers were invited to participate in the study.

### Measures

*Young Schema Questionnaire-Short Form* (*YSQ-SF*) The YSQ-SF is a 75-item scale, and is a subset of the original 205 items of the Young Schema Questionnaire-Long Form [33]. The items assess the presence of 15 early maladaptive schemas comprising emotional deprivation, abandonment, mistrust/abuse, social alienation, defectiveness, incompetence, dependency, vulnerability to harm, enmeshment, subjugation of needs, self-sacrifice, emotional inhibition, unrelenting standards, entitlement, and insufficient self-control. Each of the 75 items is rated on a six-point scale from 1 (*Completely untrue of me*) to 6 (*Describes me perfectly*) [6]. A higher score on a given subscale reflects a greater possibility of the presence of that maladaptive schema for that individual. Validity and reliability of the Persian version of the 75-item short form has been confirmed by factor analysis [34, 35].

*Cognitive Schema Activated in Sexual Context Questionnaire (CSASCQ)* The CSASCQ was developed by Nobre and Pinto-Gouveia and is a 28-item measure that assesses cognitive schemas in response to specific sexual episodes [18]. First, four common sexual problems are presented in the female version: hypoactive sexual desire disorder, orgasmic disorder, sexual pain, and subjective arousal difficulties. Participants rated the frequency of each sexual problem from 1 (*Never occurs*) to 5 (*Happens often*). Second, participants indicated the emotions aroused by the episode, checking all that apply from a list of ten emotions: worry, sadness, disillusion, fear, guilt, shame, anger, hurt, pleasure, and satisfaction. Finally, participants were instructed to focus on the most frequent situation and associated emotions, and respond to 28 self-statements reproducing Beck’s self-schemas [1], using a five-point Likert scale from 1 (*Completely false*) to 5 (*Completely true*). A factor analysis of the 28 schemas suggest a five-factor structure [18]: undesirability/rejection; incompetence; self-depreciation; difference/loneliness; and helplessness. Scores on the five domains and the total scale were calculated through the sum of the schema items with higher scores representing greater negative schema activation. Psychometric studies demonstrate adequate test–retest reliability with a four-week interval (r = 0.66) and excellent internal consistency with a Cronbach’s alpha of 0.94. Good psychometric properties of the Persian version have been reported [36].

*Socio-demographic characteristics:* These were assessed using a predefined checklist including woman’s age (years), spouse’s age (years), marriage duration (years), couple age difference (years), number of children, oldest child age (years), youngest child age (years), perception of husband’s support, strength of religious belief, and religious activities. Husband’s support, religious belief, and religious activities were assessed on a scale of 1 to 10 (e.g., for religious activities, the higher the score the more religious activities engaged in). Other demographic variables included educational status and occupation of participants and spouses, rotational working shifts for participants or spouses, long periods of husband being away from home, consanguineous marriage, having separate bedroom (from children), living with own/spouse’s family, and perceived economic status (asking about actual income tends not to be answered by Iranian participants).

### Procedure

After obtaining the necessary permits, the researchers contacted the selected health centers in Qazvin. With the coordination of the head of the comprehensive health centers, a private exam room was provided to the research team, in order to protect participant confidentiality. Eligible individuals were identified and invited to participate in the study. An explanation of the purpose of the study was given and willing participants then answered the self-report survey in a private space and returned it directly to the member of the research team present.

### Ethics

The present study was approved by the Research Council and the Ethics Committee in Biological Research of Qazvin University of Medical Sciences (code REC.1397.398). All procedures followed were in accordance with the ethical standards of the responsible committee of Qazvin University of Medical Sciences. When inviting participants, explanations were given about the importance and goals of the research, the privacy and confidentiality of collected information, the voluntary participation in the study, and the ability to leave the study at any time. Informed written consent was obtained from all participants prior to study participation.

### Statistical analysis

Data were analyzed using Statistical Package for the Social Sciences (SPSS) v. 24. Quantitatively continuous variables were calculated using means and standard deviations and categorical variables were calculated using frequencies and percentages. A uni-variable and multi-variable linear regression was used to investigate the relationship between CSASCs, EMSs, and demographic characteristics. In the uni-variable regression model, the total CSASCQ score was entered as the dependent variable and the other variables (including demographic variables) were entered as the independent variables. Variables with a significance level less than 0.05 in the uni-variable model were selected to be entered in the multi-variable model. In the multi-variable model, the stepwise method of entering variables was used. Qualitative categorical variables were coded as dummy variables for entry in the regression model. The considerations of using regression methods including normal distribution of CSASCQ score, lack of discarded data, and lack of correlation between independent variables were controlled for.

## Results

### Demographic characteristics

All participants (*N* = 260) were married women of reproductive age. The mean age of the participants was 32.48 years (SD = 6.82) and the average length of their marriage was 10.34 years (SD = 7.51). Most participants were housewives with academic education (at least B.Sc. or B.A.) reporting moderate economic status. In the univariable regression model, all demographic variables were significantly associated with CSASCQ score. Table 1 summarizes the demographic characteristics and univariable regression results.

**Table 1:** Distribution of socio-demographic variables among participants (*N* = 260) and results of univariable and multivariable logistic regression analyses considering sexual schema as dependent variable

	Range	Mean (SD)	Results of univariable linear regression analyses		
			*B*	SE	*p*
Woman’s age (years)	18–45	32.48 (6.82)	− 0.15	0.12	0.20
Spouse’s age (years)	21–57	36.60 (7.40)	0.90	0.02	0.001
Marriage duration (years)	1–33	10.34 (7.51)	2.14	0.12	0.001
Couple age difference (years) (Spouse- women age)	− 9–14	4.13	5.07	0.29	0.001
Number of children	0–5	1.15 (1.02)	16.92	1.06	0.001
Oldest child age (years)	0–30	7.24 (7.66)	2.19	0.17	0.001
Youngest child age (years)	0–24	2.74 (4.99)	2.86	0.36	0.001
Husband’s support	0–10	8.16 (2.11)	3.83	0.13	0.001
Religious belief	0–10	7.67 (2.22)	4.11	0.13	0.001
Religious activities	0–10	5.97 (2.75)	4.73	0.19	0.001

			*N* (%)	*B*	*SE*	*p*
Number of marriages		One	252 (96.9)	34.56	0.89	0.001
		Two	8 (3.1)	1		
Educational status	Woman	Academic	178 (68.5)	33.73	1.82	0.001
		Non-Academic*	82 (31.5)	1		
	Spouse	Academic	171 (65.7)	33.47	1.91	0.001
		Non-Academic	89 (34.2)	1		
Job	Woman	Housewife	145 (55.8)	36.44	2.07	0.001
		Employed	115 (44.2)	1		
	Spouse	Employed	248 (95.4)	34.47	0.95	0.001
		Unemployed	12 (4.6)	1		
Rotational working shifts	Woman	No	206 (79.2)	35.02	1.37	0.001
		Yes	54 (20.8)	1		
	Spouse	No	192(73.8)	34.36	1.58	0.001
		Yes	68 (26.2)	1		
Long period of abstinence due to husband being away from home		No	204 (78.5)	33.03	1.57	0.001
		Yes	56 (21.5)	1		
Consanguineous marriage		No	202 (77.7)	34.83	1.44	0.001
		Yes	58 (22.3)	1		
Separate bedroom		No	135 (51.9)	1		
		Yes	125 (48.1)	34.89	2.5	0.001
Living with own/spouse’s family		No	237 (91.2)	34.25	1.10	0.001
		Yes	23 (8.8)	1		
Perceived economic status		Good	110 (42.3)	30.80	1.84	0.001
		Medium	124 (47.7)	35.10	1.73	0.001
		Poor	26 (10)	1		

^*^Non-academic degree refers to having high school diploma or lower

### Early maladaptive schemas (EMSs)

The mean score of EMS was 151.45 (SD = 40.46). The highest average subscale scores were obtained for self-sacrifice (16.12; SD = 6.75) and unrelenting standards/hyper-criticalness (15.90; SD = 5.37). Given that a subscale score greater than 15 constitutes the internalization of the maladaptive schema, these results indicate the important role of these two schemas in the minds of the participants (Table 2).

**Table 2:** Distribution of early maladaptive schema (subscales and total score) among participants (*N* = 260) and results of univariable logistic regression analysis using sexual schema as dependent variable

	Acquired range	Mean (SD)	Results of univariable linear regression analysis		
			*B*	SE	*p*
Emotional deprivation	5–30	9.10 (5.39)	3.15	0.09	0.001
Abandonment	5–28	10.25 (5.23)	2.84	0.09	0.001
Mistrust/abuse	5–30	8.83 (4.96)	3.21	0.11	0.001
Social isolation	5–20	7.15 (3.31)	4.32	0.11	0.001
Defectiveness/shame	5–18	6.50 (3.08)	7.68	0.13	0.001
Failure	5–20	7.57 (3.74)	3.97	0.11	0.001
Dependence/incompleteness	5–21	7.42 (3.45)	4.02	0.13	0.001
Vulnerability to harm/illness	5–26	9.04 (4.69)	3.23	0.10	0.001
Enmeshment	5–27	8.67 (4.61)	3.23	0.12	0.001
Subjugation	5–24	10.17 (3.98)	3.07	0.09	0.001
Self-sacrifice	5–30	16.12 (6.75)	1.84	0.06	0.001
Emotional inhibition	5–30	9.99 (5.34)	2.90	0.09	0.001
Unrelenting standards	5–30	15.90 (5.37)	1.94	0.06	0.001
Entitlement	5–30	13.55 (5.43)	2.22	0.07	0.001
Insufficient self-control	5–30	11.20 (5.57)	2.59	0.09	0.001
EMS total score	75–255	151.45 (40.46)	0.22	0.005	0.001

### Cognitive schemas activated in sexual context (CSASCs)

The most common sexual problems reported by participants were hypoactive sexual desire disorder (6.9%), subjective arousal difficulties (4.2%), orgasmic disorder (4.2%), and sexual pain (2.3%). The majority of participants reported that disillusion was a predominant feeling experienced during their usual unpleasant sexual problem (33.33%). Table 3 shows the frequency of sexual problems and related emotions. In total, the average score on the CSASCQ was 34.60. Compared to other subscales, difference/loneliness had the highest mean score (Table 4).

**Table 3:** Frequency of sexual problems and related emotions for participants (*N* = 260)

	Hypoactive sexual desire	Subjective arousal difficulty	Vaginismus	Orgasmic disorder
*Frequency of sexual problems* *N (%)*				
Never happened	134 (51.5)	157 (60.4)	179 (68.8)	147 (56.5)
Seldom	56 (21.5)	46 (17.7)	46 (17.7)	56 (21.5)
Sometimes	32 (12.3)	32 (12.3)	19 (7.3)	33 (12.7)
Frequently	20 (7.7)	14 (5.4)	10 (3.8)	13 (5.0)
Happened often	18 (6.9)	11 (4.2)	6 (2.3)	11 (4.2)
*Emotion toward frequent sexual problems* *N (%)*				
Worry	2 (11.11)	1 (9.1)	0 (0)	1 (9.1)
Sadness	2 (11.11)	0 (0)	0 (0)	0
Disillusion	6 (33.33)	5 (45.5)	4 (66.7)	5 (45.5)
Fear	0 (0)	0 (0)	0 (0)	0
Guilt	2 (11.11)	2 (18.2)	0 (0)	0
Shame	0 (0)	0 (0)	0 (0)	0
Anger	0 (0)	0 (0)	0 (0)	0
Hurt	1 (5.6)	1 (9.1)	0 (0)	0
Pleasure	2 (11.11)	2 (18.2)	2 (33.3)	2 (18.2)
Satisfaction	3 (16.7)	0 (0)	0 (0)	3 (27.3)
Total	18 (100)	11 (100)	6 (100)	11 (100)

**Table 4:** Sexual schema subscale and total mean scores

Sexual schema (range)	Mean (SD)	Corrected mean* (SD)
Undesirability/rejection (5–25)	6.33 (2.77)	1.27 (0.55)
Incompetence (9–45)	12.33 (4.58)	1.23 (0.46)
Self-depreciation (4–20)	5.17 (2.45)	1.29 (0.61)
Difference/loneliness (3–15)	5.04 (2.23)	1.68 (0.74)
Helplessness (4–20)	5.70 (2.87)	1.43 (0.72)
Total (25–125)	34.60 (12.59)	1.38 (0.50)

^*^Mean score of each subscale was calculated by summing the items based on the original instruction. Due to unequal number of items in each subscale, mean of each subscale was corrected by dividing the sum scores by the number of items for each subscale. This correction was done to have comparable scores across subscales

The relationship between CSASCs and EMSs was examined. The univariable regression models considering CSASCQ score as the dependent variable and EMS subscales as independent variables showed that all EMS subscales had a significant relationship with CSASCs among married women of childbearing age (Table 2). To examine this relationship more accurately, a multivariate linear regression was performed.

In the multivariate regression model, EMSs and demographic variables were entered using the stepwise function (Table 5). The results of the multivariate model showed that three facets of EMS were significant independent variables in predicting CSASCs: emotional deprivation (β = 0.28, *p* < 0.001), social isolation (β = 0.31, *p* < 0.001) and emotional inhibition (β = 0.14, *p* < 0.001). The mean scores on the CSASCQ increased by 1.47, 0.98 and 0.47 points for each point increase in the schemas of social isolation, emotional deprivation and emotional inhibition, respectively.

**Table 5:** Predictors of sexual schema according to multivariable linear regression analysis

	Unstandardized coefficients		Standardized coefficients	*p-* value	Collinearity statistics	
	*B* (95% CI)	SE	Beta		Tolerance	VIF
Job	5.44 (2.90–7.97)	1.29	.11	< .001	.43	2.33
Consanguineous Marriage	8.41 (5.70–11.11)	1.37	.20	< .001	.27	3.70
Emotional deprivation	0.98 (0.71–1.24)	0.13	.28	< .001	.20	5.04
Social isolation	1.47 (1.05–1.89)	0.21	.31	< .001	.14	7.17
Emotional inhibition	0.47 (0.21–0.72)	0.13	.14	< .001	.18	5.42
Model Summary	R^2^ = 0.93; Adjusted R^2^ = 0.92; Durbin-Watson = 2.09; F = 396.85; *p* < 0.001					

*CI* Confidence interval, *VIF* Variance inflation factor

Occupation (β = 0.11, *p* < 0.001) and consanguineous marriage (β = 0.20, *p* < 0.001) were the only demographic variables that remained significant in the multivariate regression model. The mean score of CSASCQ for housewives was 5.44 points higher than that of employed women; the score was 8.41 points higher for women in non-consanguineous marriage compared to those in consanguineous marriage. In total, the multivariate model explained 92% of the variance of CSASCQ score.

## Discussion

The present study investigated the relationship between early maladaptive schemas (EMSs) and cognitive schemas activated in the sexual context (CSASCs) among married women of childbearing age. Self-sacrifice and unrelenting standards were two maladaptive EMSs and the difference/loneliness subscale was the main cognitive schema activated in the sexual context among participants. Further multivariate analysis demonstrated that three early maladaptive schemas of emotional deprivation, social isolation, and emotional inhibition schemas predicted CSASCs.

The highest average subscale scores on the early maladaptive schemas were related to self-sacrifice and unrelenting standards, indicating that these two schemas played important roles in the cognition of the participants. In Iranian culture, self-sacrifice is valued among women. From early childhood, Iranian girls are acquainted with this feature by their mothers and follow their example; later in life, self-sacrifice is seen as one of the values of femininity and motherhood. The spirit of self-sacrifice is also highly emphasized in Iranian Islamic culture and is emphasized in women. In Iranian culture, mothers are encouraged to devote more time to family and children, and may experience negative emotions about themselves if unable to perform these tasks fully. Therefore, due to the fact that women have many responsibilities and helping others is a priority for them, unrelenting standards may be emphasized by them. In previous Iranian studies, the self-sacrifice and unrelenting schemas have also been identified as maladaptive schemas among Iranian women [37, 38].

The study further showed that participants’ average CSASCQ score was 34.6 (out of 125), with higher scores indicating stronger problematic cognitive schemas. The difference/loneliness subscale had the highest mean score, demonstrating that participants’ perceived level of difference from partner or loneliness most impacted their cognitive schemas activated in sexual context. The most predominant feeling associated with hypoactive sexual desire was disillusion. In the previous study, the mean score of cognitive schemas relating to sexual problems on the CSASCQ was 35.56 (SD = 14.68) with the highest mean score also on the difference/loneliness subscale [23]. Other Iranian studies did not use the CSASCQ questionnaire. A study by Piexeto found that among women with sexual problems, feelings of difference from partner/loneliness and undesirability/rejection schemas were more common in sexual situations than among healthy women [28].

The multivariate analysis demonstrated that three early maladaptive schemas predicted CSASCs: emotional deprivation, social isolation, and emotional inhabitation. Various studies have shown that EMSs play an important role in predicting the outcomes of couples’ sexual and marital relationships. Hamidi et al. [39] reported that women with vaginismus had more EMSs and more anxious attachment styles than healthy women. In a study by Khosravi et al. [40], feelings of shame/defectiveness, emotional deprivation, and mistrust/abuse were the most common schemas among women experiencing domestic violence in the context of marital conflict. Moreover, Esmaili et al. [38] found that the presence of higher emotional deprivation and unrelenting standards/hypocrisy were predictors of lower marital satisfaction. Three schemas significantly predicted higher SCASCs (i.e., emotional emotion, emotional deprivation, and social isolation).

The findings suggest that individuals with emotional inhibition schemas cannot express their feelings and emotions spontaneously. It is difficult for them to say that they love and value others. They believe that it is better to hide their emotions and to control themselves [5]. Therefore, being emotionally inhibited could make sexual activity challenging. The inability by individuals to express themselves, to let go of inhibitions impact on the ability to be relaxed enough physically to enjoy sexual activity and to reach orgasm. Another schema related to CSASCs was emotional deprivation. Individuals with emotional deprivation schemas feel that no-one wants or can meet their needs. They often think that others do not really listen to them and do not understand them. They may avoid romantic relationships or have relationships that are short-lived [2]. One of the requirements of a good sex life is to have a dyadic relationship and a sense of trust in the sexual partner to assess sexual needs. Given that these individuals experience emotional deprivation, they may have difficulty in sexual communication and having a desirable sexual relationship. Individuals with social isolation schemas may experience anxiety in new situations and therefore avoid participating in such situations. They feel that they are not like other individuals and they may get nervous about being with new people and they really do not know what to talk to them about. They may also become anxious and worried about how they will feel about themselves [5]. When they are anxious, they cannot use their social skills and therefore feel isolated and insecure. While they need a closer relationship with others, they may not be able to do so and this may lead to problems in initiating sexual relationships. Therefore, it may be helpful to explore these three specific early maladaptive schemas among women who are having difficulty in their sexual relationships.

Other research [41] has shown that individuals with sexual dysfunction experience significantly more negative schemas when exposed to unsuccessful sexual situations than sexually healthy individuals. Most men and women with sexual problems interpret their negative sexual events within the incompetence self-schema (e.g., *“I’m powerless”, “I’m incompetent”*). These findings are consistent with recent research showing that individuals with sexual dysfunction tend to make internal, persistent, and universal attributions to negative sexual experiences. Therefore, it is clear that defective cognitive structures provide a basis for sexual dysfunction. This highlights the importance of using cognitive theory to develop therapeutic models and approaches to improve cognitive schemas activated in a sexual context and marital satisfaction [41].

Occupation and consanguineous marriage were important predictors of CSASCQ score in the present study. The results showing an association between these variables with sexually-related issues (e.g., sexual satisfaction) is contradictory; while higher rates of female sexual dysfunction [42] and less sexual satisfaction [43] are reported among women in consanguineous marriage, Salehiniya, et al. [44] did not find any significant association between these variables.

### Limitations and strengths

When interpreting the findings of the present study, the following limitations should be considered. The cross-sectional design does not allow the determination of causal relationships. Further limitations include the self-report method, and the relatively limited sampling in comprehensive health centers. The exclusion of unmarried women in the present study was due to cultural reasons. Based on Iranian and Islamic culture, individuals can only commence their sexual relationship once they are married. Because the present study was conducted in the context of Iranian society, only married women were recruited as participants. Therefore, the results cannot be generalized to unmarried sexually active women. Despite these limitations, this is the first study to investigate the relationship between early maladaptive schemas and cognitive schemas activated in a sexual context among married women of childbearing age. The use of univariable as well as multivariable linear regression models made it possible to examine this relationship more thoroughly. Further studies are needed in order to test the role of cognitive schemas activated among women with specific sexual disorders (e.g., genito-pelvic pain disorder).

## Conclusion

Early maladaptive schemas of emotional deprivation, social isolation, and emotional inhibition were significantly associated with cognitive schemas activated in the sexual context. Therefore, in premarital and marital counseling relating to sexual issues, the study of schemas and the integration of schema therapy and cognitive therapy should be considered.

## Data Availability

The datasets used and/or analyzed during the current study are available from the corresponding author upon reasonable request.

## Abbreviations

CSASCs: Cognitive schemas activated in the sexual context
EMSs: Early maladaptive schemas
